# The Importance of the Pars Interarticularis as a Landmark for Safe Lumbar Pedicle Screw Placement: Technical Note

**DOI:** 10.7759/cureus.4413

**Published:** 2019-04-09

**Authors:** William Clifton, David Williams, Aaron Damon, Conrad Dove, Mark Pichelmann

**Affiliations:** 1 Neurosurgery, Mayo Clinic, Jacksonville, USA; 2 Internal Medicine, Marine Corps Air Station Beaufort, Beaufort, USA; 3 Neurosurgery, Mayo Clinic, Rochester, USA

**Keywords:** spine, fusion, pedicle screw, navigation, pars, anatomy

## Abstract

The use of navigational adjuncts for pedicle screw placement has increased in popularity among surgeons with access to this technology. However, it remains important to have a comprehensive understanding of posterior bony element anatomy with respect to the location of the pedicle in order to ensure safe placement of pedicle screws. Proper exposure and identification of the pars interarticularis provide a helpful landmark during pedicle screw placement in order to confirm navigation accuracy and avoid misplaced instrumentation. In this technical note, we highlight the surgical anatomy of the pars interarticularis of the lumbar spine and its relationship to the lateral, inferior, and medial borders of the pedicle using diagrams and cadaveric dissections.

## Introduction

There are several methods described for safe and accurate placement of pedicle screws in the lumbar spine [1]. The use of navigational adjuncts for pedicle screw placement has increased in popularity among surgeons with access to this technology [2-5]. One disadvantage of this method is the requirement of the surgeon to be looking away from the operative field during pedicle access and screw placement. Errors in software registration may go undetected by the operator if the surgeon is not continuously correlating the navigational screen with direct visualization of intraoperative anatomy [6-7]. This may result in erroneous instrumentation with the risk of neurologic injury. It is important to have a comprehensive understanding of posterior bony element anatomy with respect to the location of the pedicle in order to ensure safe placement of pedicle screws. In this manuscript, we highlight the surgical anatomy of the pars interarticularis of the lumbar spine and its relationship to the lateral, inferior, and medial borders of the pedicle using diagrams and cadaveric dissections. Proper exposure and identification of the pars provide a helpful landmark during pedicle screw placement in order to confirm navigation accuracy and avoid misplaced instrumentation.

## Technical report

**Figure 1:**
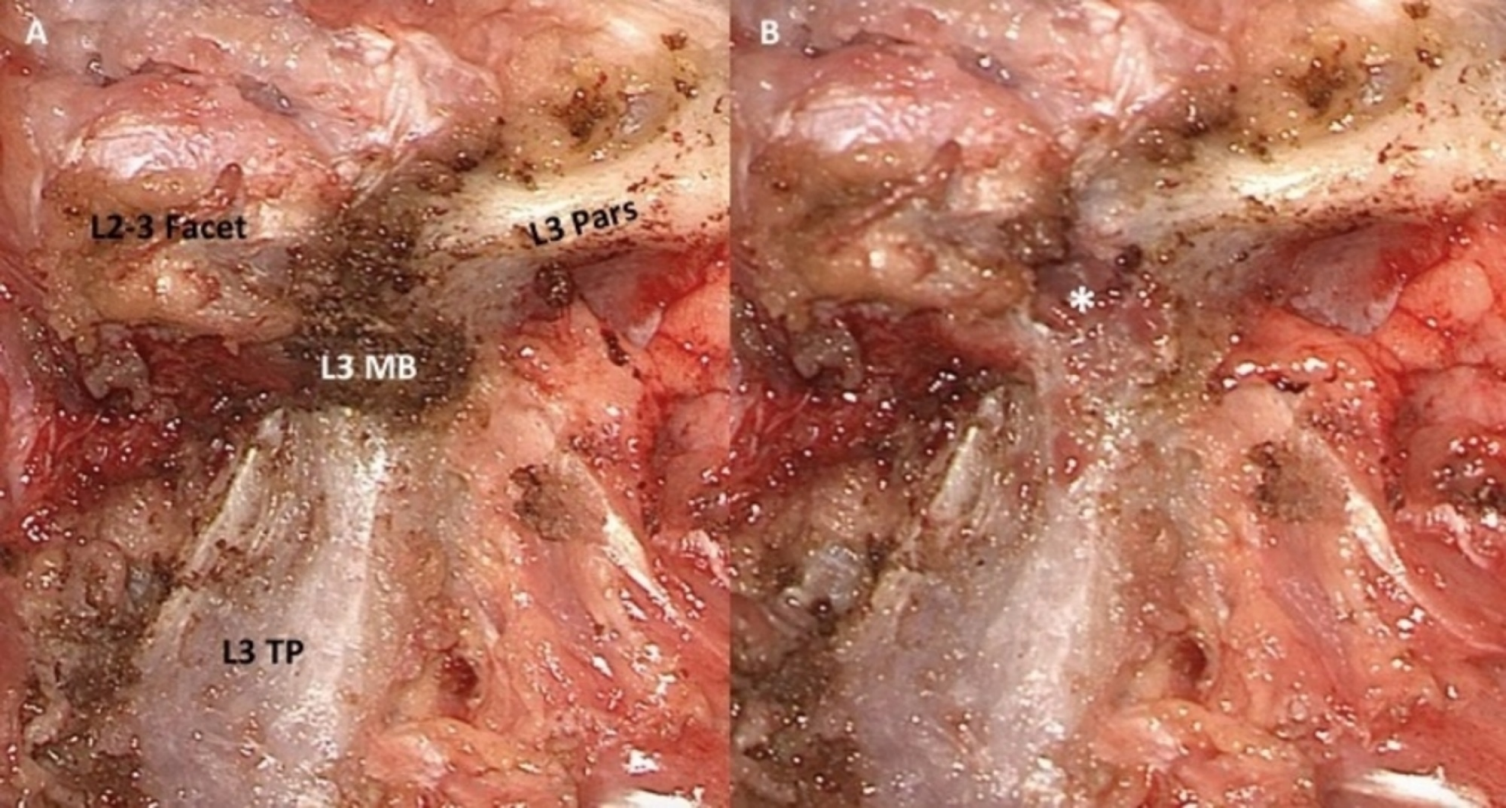
Exposure of the posterior elements of the lumbar spine Locating the pedicle. (A) The junction of the pars, transverse process (TP) and superior facet is shown. The mammillary body (MB) is exposed, and is a reliable landmark for entry into the pedicle. It is important to remove all soft tissue at this junction in order to determine the pedicle boundaries. (B) When the mammillary process is removed, the cancellous bone (asterisk) continuous with the pedicle is revealed.

The pars interarticularis is the lateral portion of the posterior bony elements that connects the inferior and superior facets at each lumbar level. The pars is continuous with the unilateral lamina, superior facet, inferior facet, transverse process, and pedicle [8-9]. The junction of the pars with the superior facet and midpoint of the transverse process is a previously described reliable landmark for entry point into the midportion of the lumbar pedicle [10]. Often, there is a bony prominence at this junction that aids identification known as the mammillary process (Figure 1). The detailed anatomy of the pars can be used as a visual anatomic landmark to estimate the pedicle boundaries and avoid breaches during screw placement [11-12].

**Figure 2:**
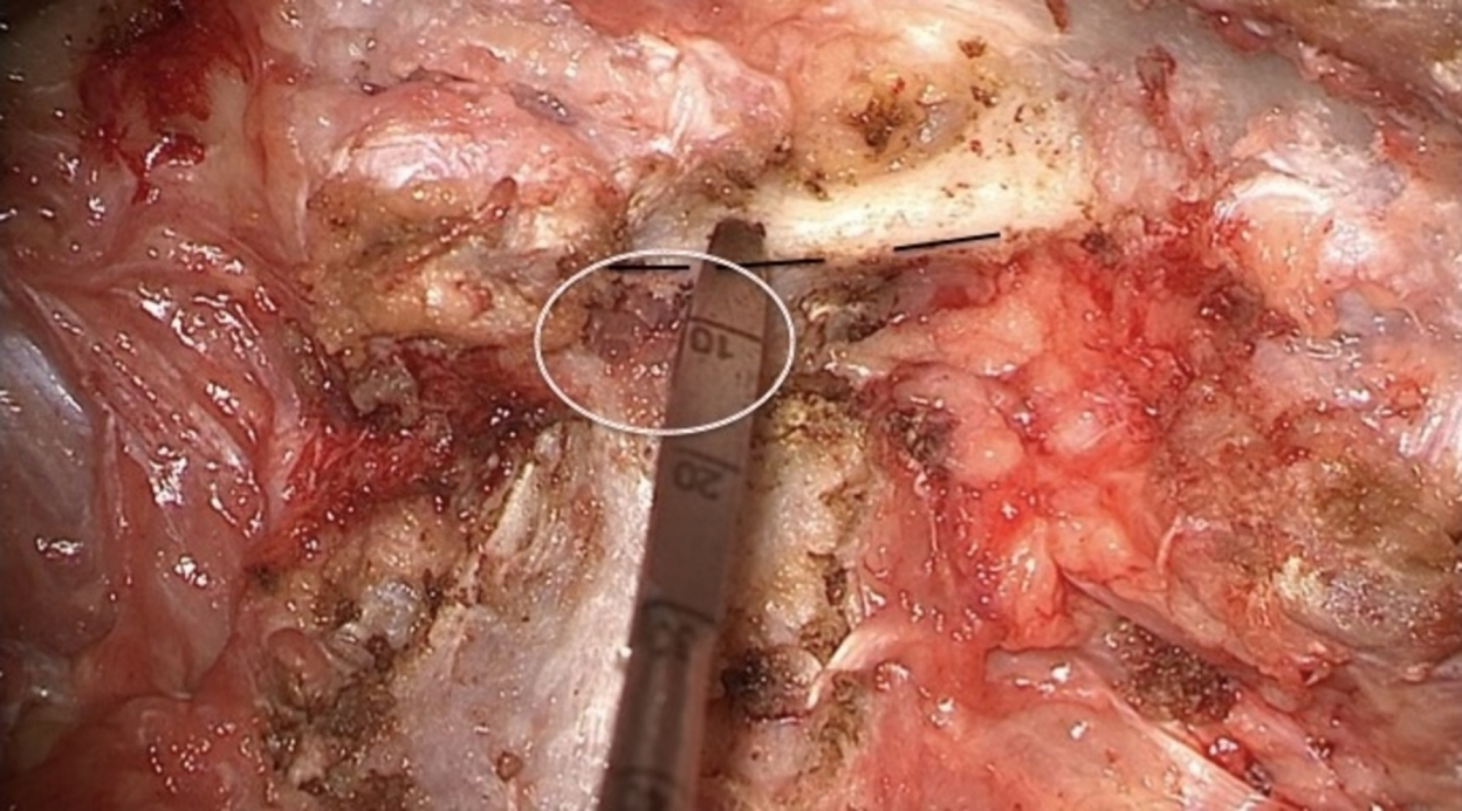
Approximating the medial border of the pedicle The white circle indicates the approximate diameter and location of the pedicle in relation to the posterior elements. The black dashed line highlights the lateral border of the pars interarticularis. Screw insertion at or medial to this point (pedicle probe) has a high risk of breach into the lateral recess and spinal canal.

The lateral border of the pars creates the bony roof just inferior to the lateral recess and superior to the intervertebral foramen in the lumbar spine at each level [13-14]. The medial border of the pedicle can be estimated by complete exposure of the lateral border of the pars with respect to the lamina and superior facet (Figure 2). Screw starting point at or medial to this point increases the risk of a medial breach into the lateral recess. Proper exposure of this border also allows surgeons to gauge the accuracy of navigational instruments during screw trajectory planning. 

**Figure 3:**
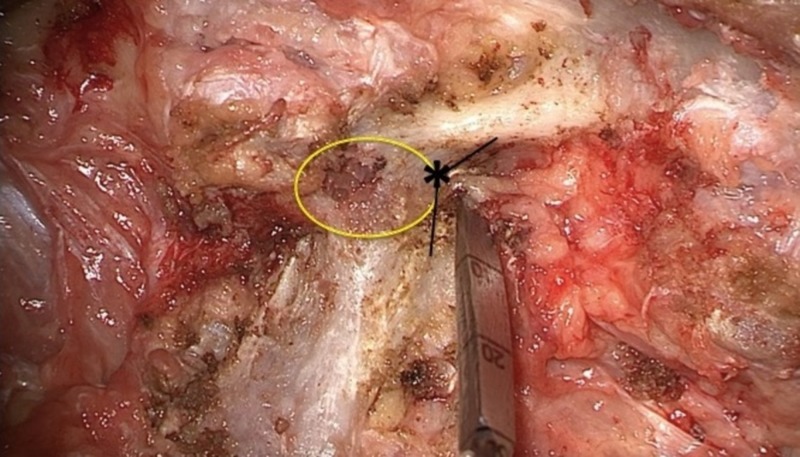
Approximating the inferior border of the pedicle The yellow circle indicates the approximate diameter and location of the pedicle in relation to the posterior elements. The inferior-most junction of the transverse process and lateral pars estimates the inferior portion of the pedicle (black lines, asterisk). The exiting nerve root is at risk with screw insertion or trajectory inferior to this point.

The junction of the pars with the proximal and inferior portion of the transverse process is a reliable landmark for the inferior border of the pedicle wall. It is important to remove all soft tissue and expose this junction completely in order to estimate the location of the superior portion of the foramen and the exiting nerve root (Figure 3). Complete exposure of the junction of the pars and superior facet lessens the risk of an inferior breach due to direct visualization of the inferior pedicle boundary.

**Figure 4:**
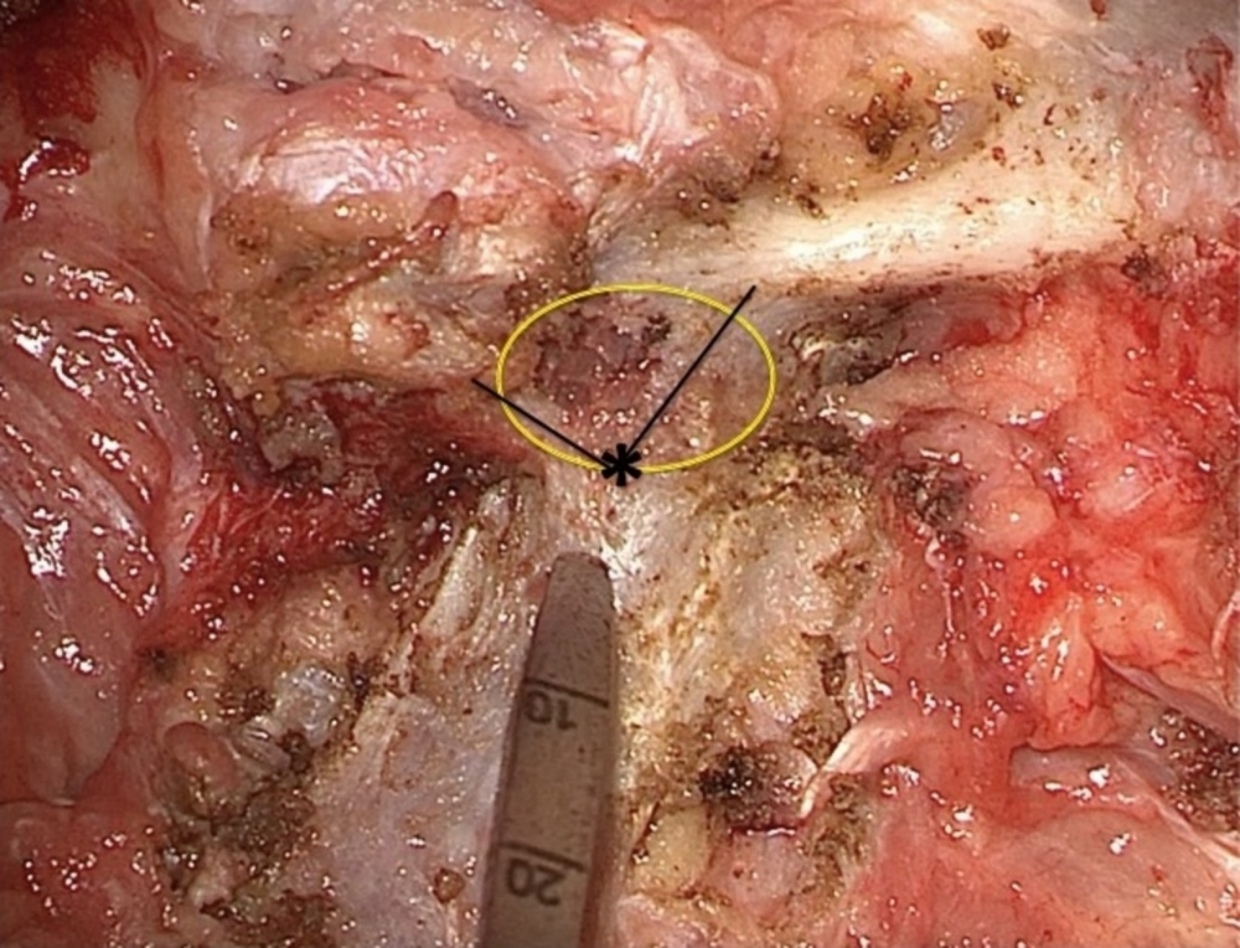
Approximating the lateral boundary of the pedicle The yellow circle indicates the approximate diameter and location of the pedicle in relation to the posterior elements. The lateral junction of the superior facet and pars meets the transverse process and estimates the lateral boundary of the pedicle (black lines, asterisk). Starting lateral to this point (pedicle probe) increases the risk of a lateral breach and insertion of the pedicle screw through the ventral cortex of the transverse process into the psoas muscle.

The lateral junction of the pars, superior facet, and transverse process is a reliable landmark for the lateral boundary of the pedicle. The bony surface of the superior facet transitions into the transverse process laterally, and joins the pars at the inferior-most portion (Figure 4). The lateral border of the pedicle can be estimated by complete exposure of this junction. Screw starting point lateral to the lateral portion of the superior facet and pars junction increases the risk of a lateral breach through the inferior cortical surface of the transverse process with a “straight down” trajectory. Increasing the medial angulation of the screw trajectory with a lateral starting point may allow for complete insertion into the pedicle; however, there may be a significant discrepancy in screw alignment with adjacent levels. This may drastically increase the difficulty of rod placement, especially with long constructs.

## Discussion

**Figure 5:**
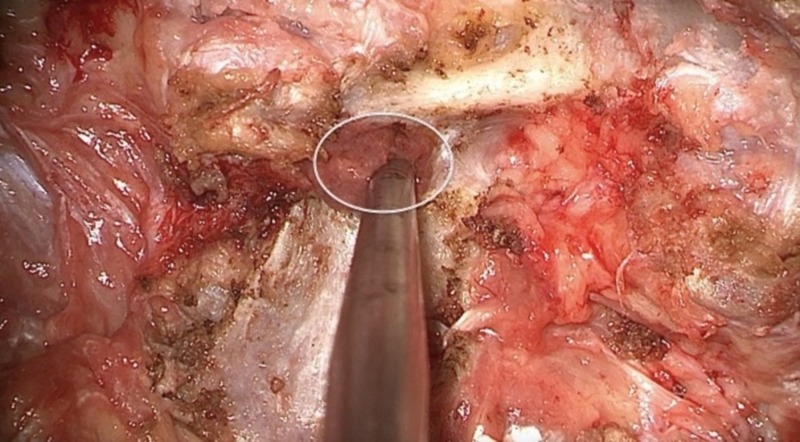
Accessing the midpoint of the pedicle The white circle indicates the approximate diameter and location of the pedicle in relation to the posterior elements. Through complete exposure of the pars and defining the pedicle boundaries as previously described, the cancellous bone of the pedicle is revealed after removal of the mammillary process and accessed with a curved probe.

**Figure 6:**
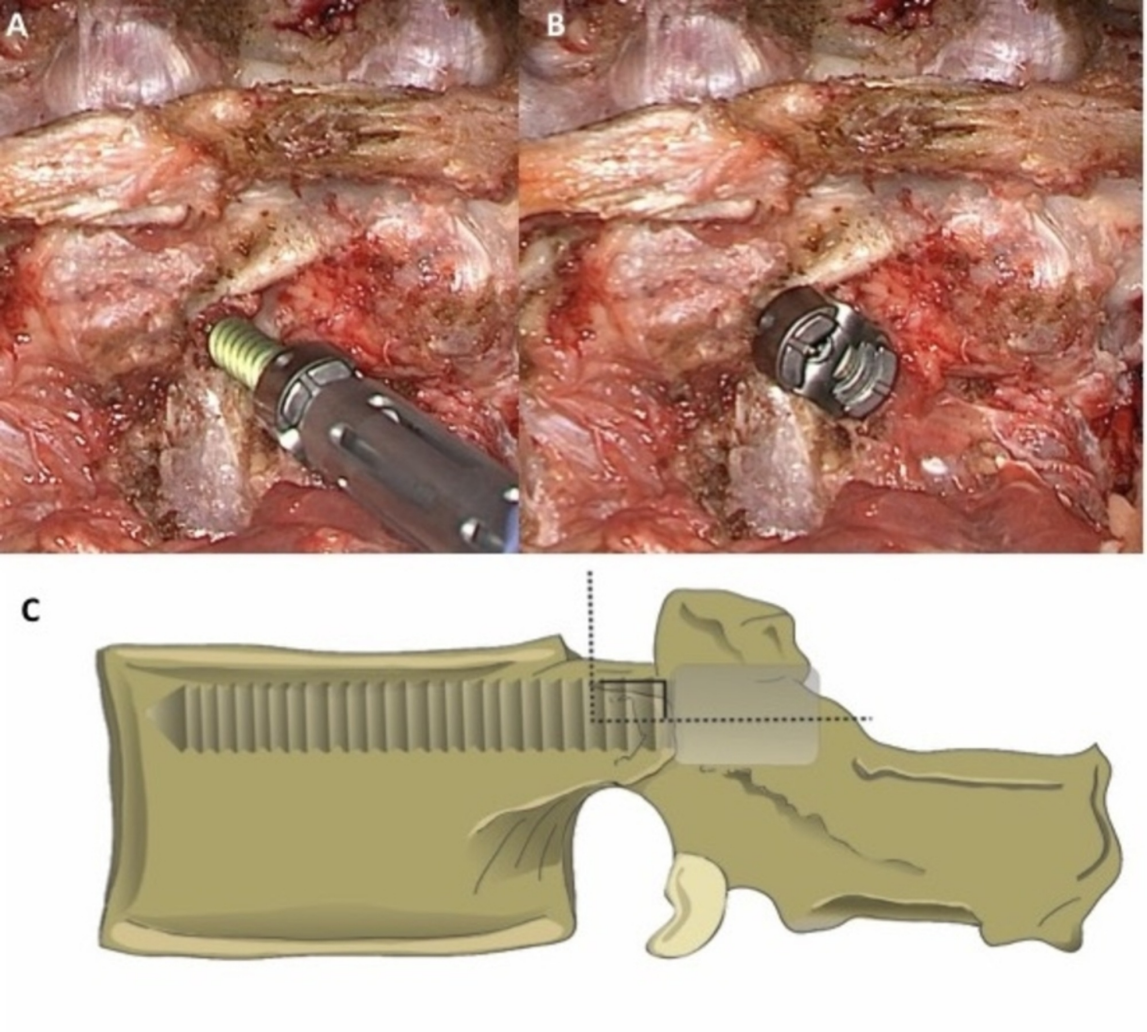
Proper pedicle screw insertion (A) It is important to be aware of the screw insertion trajectory and (B) head position in relation to the previously estimated pedicle boundaries with respect to the pars in order to confirm proper placement into the pedicle. Excessively angled trajectories or extreme screw head position in the medial-lateral plane should raise question on the accuracy of screw placement. (C) The cranial-caudal angle of screw insertion should be perpendicular to the transverse process.

Through proper exposure and identification of the pars, the midpoint of the pedicle for proper screw starting point and placement can be accurately determined. The mammillary process is first removed with a rongeur or drill. The cancellous bone continuous with the pedicle is revealed. The pedicle is then accessed with a curved probe (Figure 5). Screw placement should correspond to the previously selected entry point. The direction of screw insertion, as well as the position of the screw head during and after placement, should be contained within the previously defined pedicle boundaries by the exposed pars interarticularis (Figure 6). Any deviation from this expected position should alert the operator that the screw might not be placed optimally within the pedicle.

## Conclusions

The pars interarticularis is a key element in determining the lateral, inferior, and medial borders of the lumbar pedicle. Complete exposure of this structure is advised prior to screw placement in order to define the midpoint of the pedicle for a proper screw starting point. With the increasing use of computed tomography navigation and robotic-assisted techniques, it remains important for the surgeon to have a comprehensive understanding of spinal anatomy in order to verify the accuracy of the navigational systems and avoid complications from misplaced instrumentation.
